# Enhanced Rail Surface Defect Segmentation Using Polarization Imaging and Dual-Stream Feature Fusion

**DOI:** 10.3390/s25113546

**Published:** 2025-06-04

**Authors:** Yucheng Pan, Jiasi Chen, Peiwen Wu, Hongsheng Zhong, Zihao Deng, Daozong Sun

**Affiliations:** College of Electronic Engineering & College of Artificial Intelligence, South China Agricultural University, Guangzhou 510642, China; pyc@stu.scau.edu.cn (Y.P.); 20223169001@stu.scau.edu.cn (J.C.); pven@stu.scau.edu.cn (P.W.); zhonghs@stu.scau.edu.cn (H.Z.); 20233169011@stu.scau.edu.cn (Z.D.)

**Keywords:** rail surface defect detection, polarization imaging, defect segmentation, deep learning, feature fusion networks, DeepLabV3+

## Abstract

Rail surface defects pose significant risks to the operational efficiency and safety of industrial equipment. Traditional visual defect detection methods typically rely on high-quality RGB images; however, they struggle in low-light conditions due to small, low-contrast defects that blend into complex backgrounds. Therefore, this paper proposes a novel defect segmentation method leveraging a dual-stream feature fusion network that combines polarization images with DeepLabV3+. The approach utilizes the pruned MobileNetV3 as the backbone network, incorporating a coordinate attention mechanism for feature extraction. This reduces the number of model parameters and enhances computational efficiency. The dual-stream module implements cascade and addition strategies to effectively merge shallow and deep features from both the original and polarization images. This enhances the detection of low-contrast defects in complex backgrounds. Furthermore, the CBAM is integrated into the decoding area to refine feature fusion and mitigate the issue of missing small-target defects. Experimental results demonstrate that the enhanced DeepLabV3+ model outperforms existing models such as U-Net, PSPNet, and the original DeepLabV3+ in terms of MIoU and MPA metrics, achieving 73.00% and 80.59%, respectively. The comprehensive detection accuracy reaches 97.82%, meeting the demanding requirements for effective rail surface defect detection.

## 1. Introduction

Rails are critical components in industrial manufacturing and are widely used in automation equipment, machine tools, robots, and precision instruments designed for high-precision linear motion. Typically, rails feature a composite structure, primarily composed of high-quality carbon steel or alloy steel. This material selection ensures the rails exhibit superior strength, hardness, and wear resistance [1]. However, as the demand for industrial automation and high-precision manufacturing continues to rise, the requirements for rail surface quality have become increasingly stringent. Defects such as scratches, pits, and cracks can compromise the operating accuracy and service life of equipment, potentially leading to production failures and safety risks [1,2]. While traditional manual inspection methods can detect these defects to a certain extent, their efficiency and accuracy are often insufficient to meet the growing demands of modern manufacturing [1]. Consequently, the development of efficient and precise rail surface defect detection technologies has become essential to maintaining the performance and quality of industrial operations.

The rise of deep learning technology has spurred the development of semantic segmentation techniques, which have become essential for analyzing industrial surface defect images. Semantic segmentation-based surface defect detection methods provide a promising solution for the efficient and precise identification of defects on rails. Numerous defect detection methods have been proposed in existing research. For instance, Yu et al. [2] introduced a two-stage deep learning method for surface defect detection that integrates segmentation and detection stages using a fully convolutional network (FCN) for image processing. However, the large receptive field of this method can lead to the loss of local details, affecting the detection accuracy for small defects. In contrast, Gang et al. [3] enhanced YOLOv8 by incorporating the CBAM-RFB module and multi-scale feature fusion, improving both the accuracy and speed of steel surface defect detection. Despite these advancements, challenges remain in detecting small targets and adapting to complex backgrounds. Li et al. [4] proposed a multi-scale residual attention network to address the challenges posed by limited defect features and small sample sizes of light guide plates. However, the attention mechanism struggles to adequately activate features of tiny defects, leading to potential missed detections or misjudgments, particularly when complex background noise is present. In another study, Wu et al. [5] developed a hybrid deep learning architecture for rail surface segmentation and defect detection, but its lightweight design limits model capacity and impairs feature learning for small defects. Furthermore, defects often occupy a minimal proportion of image pixels and have a low signal-to-noise ratio. The low contrast between textured backgrounds and defect regions further complicates detection, and environmental factors such as lighting continue to hinder the effectiveness of defect detection methods [6].

Polarization images, as a novel information source, present new opportunities for defect detection through deep learning. Compared to traditional RGB images, polarization images capture additional characteristics that reflect the features of an object’s surface, microstructure, and material properties, providing crucial information not detectable by conventional RGB images. This unique capability is particularly valuable for the accurate identification and localization of small defects. The integration of polarization images into deep learning-based defect detection, leveraging deep learning’s powerful feature extraction and pattern recognition abilities, can improve the accuracy and efficiency of detection. This approach offers robust support for quality control and automated inspection in industrial production, making it highly promising for widespread application and research. For instance, Ding et al. [7] significantly improved the detection accuracy and sensitivity for surface micro defects in curved optical components by incorporating transmission polarization detection and multi-parameter analysis. Similarly, Deng et al. [8] enhanced surface defect segmentation in transparent materials by capturing images from multiple polarization angles. Additionally, Chen et al. [9] boosted defect detection in reflective materials by employing dilated separable convolutions in conjunction with an encoder–decoder architecture. However, these methods still face challenges in accurately segmenting the edges of tiny defects, and the substantial computational load of these models restricts their applicability in real-time or large-scale industrial inspection scenarios.

To further enhance the precision of surface defect segmentation on rails, this paper proposes a dual-stream feature fusion DeepLabv3+ network that integrates light-intensity images and polarization information to detect small defects with low contrast in complex backgrounds. The network independently extracts light intensity and linear polarization features from the polarization images and effectively fuses them, ensuring that the contrast between the target defect and the background is preserved. The MobilenetV3-CA backbone network is employed to optimize the model’s efficiency, while the CBAM is integrated to improve the model’s ability to capture critical information. This combination of techniques enables highly accurate segmentation of small-target defects. The contributions of this paper can be summarized in the following three points:

1. We introduce a dual-stream network architecture that integrates light-intensity and polarization images for enhanced defect detection on rail surfaces. This architecture independently extracts and combines features from both modalities, improving contrast and detail visibility in low-contrast and complex environments.
2. Our approach incorporates polarization imaging into a DL framework, utilizing polarization-specific data to significantly enhance the detection of small-scale and low-contrast defects. This integration extends the capabilities of traditional RGB-based defect detection systems.
3. We utilize a pruned MobileNetV3 backbone enhanced with coordinate attention for efficient feature extraction, complemented by the Convolutional Block Attention Module for focused feature refinement. This combination optimizes the model for high accuracy and efficiency, making it suitable for real-time industrial applications.
4. Extensive experiments on real-world ratio surface defect segmentation datasets demonstrate the effectiveness and superiority of our proposed method.

## 2. Related Work

The detection and analysis of surface defects on industrial rails is a critical area of research due to the significant impact of such defects on operational efficiency and safety. Early approaches primarily relied on manual inspection methods, which, although somewhat effective, do not meet the efficiency and accuracy requirements of modern manufacturing systems. As the demand for higher precision and automation grows, there is a significant shift towards employing advanced computational techniques, particularly deep learning, to enhance defect detection capabilities.

### 2.1. Deep Learning in Defect Detection

The application of deep learning to rail surface defect detection has undergone significant transformation in recent years, progressing from basic classification models to sophisticated detection and segmentation architectures. Initial approaches in industrial defect detection predominantly relied on traditional machine vision techniques that utilized manually designed features such as texture, color, and shape characteristics [10]. These methods, while computationally efficient, struggled with the inherent variability of rail surface defects and were particularly sensitive to environmental factors like lighting conditions and surface contamination [11].

The advent of convolutional neural networks (CNNs) marked a paradigm shift in defect detection capabilities. Early CNN-based approaches focused primarily on defect classification, with notable work [12] employing a joint detection CNN architecture that achieved remarkable results. However, these initial implementations lacked precise localization capabilities, a critical requirement for rail maintenance applications [13]. The development of two-stage detection frameworks, particularly variants of Faster R-CNN, addressed this limitation by combining region proposal networks with classification heads. Du et al. demonstrated the effectiveness of this approach for rail defect detection, achieving high accuracy through optimized anchor box functions and multi-scale feature pyramids [14]. Recent advancements have seen the dominance of single-stage detectors, particularly the YOLO series, in rail defect detection applications. Bai et al. [15] enhanced YOLOX by integrating BiFPN for feature fusion and incorporating the NAM attention mechanism. The YOLO series has further pushed performance boundaries through architectural innovations like decoupled heads and distribution fusion loss function [16]. Despite these advancements, significant challenges persist in detecting small-scale defects and maintaining performance in complex operational environments. Han et al. [17] attempted to address the small defect problem through multi-scale residual attention networks, while Zhang et al. [18] proposed a low-pass U-Net architecture that incorporated adaptive variance Gaussian filters to preserve high-frequency defect information in steel strips.

### 2.2. Polarization Imaging

Polarization imaging has emerged as a transformative approach for rail surface inspection, providing critical material property data that conventional RGB imaging cannot capture. Recent advancements in Stokes parameter imaging and Mueller matrix polarimetry have enabled unprecedented characterization of surface microstructures and stress patterns. This modality has proven particularly effective for detecting micro-cracks and early-stage rolling contact fatigue (RCF) defects that are invisible to traditional vision systems.

Ref. [19] demonstrated significant improvements in micro-defect detection for high-speed rail components, achieving a 92.3% detection rate polarization imaging system. Their research highlighted how polarized light interacts differently with surface stress concentrations compared to normal surface features, providing a unique fingerprint for early defect identification. Similarly, Luo et al. [20] developed a snapshot polarization camera system specifically for rail inspection that captures full Stokes vectors in a single exposure, enabling real-time acquisition while maintaining resolution. Recent breakthroughs in computational polarization imaging have addressed several key challenges in industrial applications. The polarization feature enhancement network [21] uses a physics-informed deep learning approach to reconstruct high-quality polarization images from limited measurements, reducing acquisition time while maintaining defect detection accuracy. The integration of polarization data with deep learning presents unique computational challenges due to the high-dimensional nature of polarimetric data. To address this, Ref. [22] developed a lightweight architecture that processes polarization features through separate branches before fusing them with spatial information. Their approach reduced inference time while improving small defect detection rates. Despite these advancements, three key challenges remain in polarization-based rail inspection: (1) The high cost [23] and complexity of industrial-grade polarization cameras and (2) real-time processing requirements for high-speed inspection systems. Our proposed dual-stream architecture addresses these limitations by combining efficient polarization feature extraction using MobileNetV3-CA with attention-based fusion via the CBAM. This approach builds on recent work in computational polarization while introducing novel optimizations for rail-specific defect detection.

## 3. Improvement of the DeepLabv3+ Network

**Figure 1:**
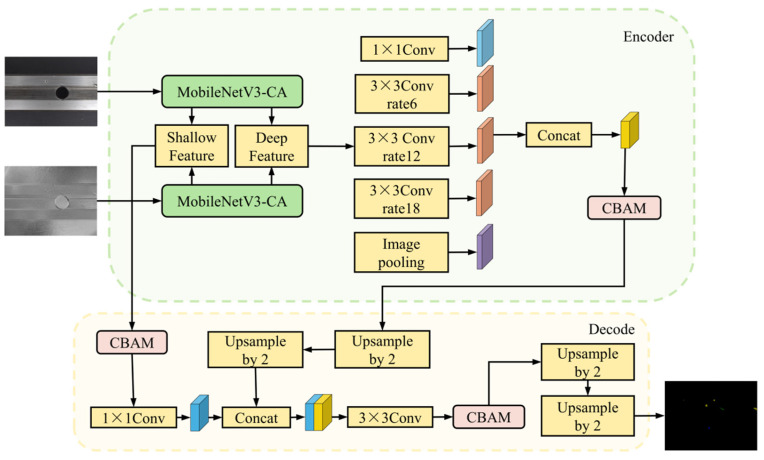
Improved DeepLabv3+ model structure. The network uses MobileNetV3-CA for multi-modal feature extraction and integrates CBAMs into both the encoder and the decoder to enhance feature representation, ultimately generating the segmentation output.

In segmenting surface defects on rails, traditional models often encounter challenges such as high rates of missed detections, false positives, and uneven edge segmentation. This paper presents an enhanced DeepLabv3+ [12] network structure that improves accuracy and detail in defect segmentation, as shown in Figure 1. The improvement leverages the DeepLabv3+ framework to optimize robustness in complex scenarios through comprehensive adjustments.

The first enhancement involves using the pruned MobileNetV3 network [24] as the backbone in conjunction with the coordinate attention mechanism [25] during the encoding stage. MobileNetV3 is selected for its strong feature extraction capabilities while maintaining computational efficiency. The coordinate attention mechanism improves the spatial feature representation, providing significant advantages in defect localization.

The second enhancement occurs in the decoding stage, where a multi-scale feature fusion module is designed to enhance the model’s capacity to detect defects at varying scales. In this approach, two backbone networks are employed to extract both high-level and low-level features from the input image. These features are then concatenated directly, preserving image information while fusing semantic information at different levels.

The third improvement integrates the CBAM [26] into the ASPP module and the shallow feature extraction network in the encoder during the decoding stage. The CBAM applies attention mechanisms across both the channel and spatial dimensions, enhancing the model’s ability to emphasize important features while suppressing irrelevant ones. Consequently, the model exhibits superior performance in accurately identifying and segmenting defect regions, particularly those that are small and challenging to detect.

### 3.1. MobileNetV3-CA Backbone Module

**Figure 2:**
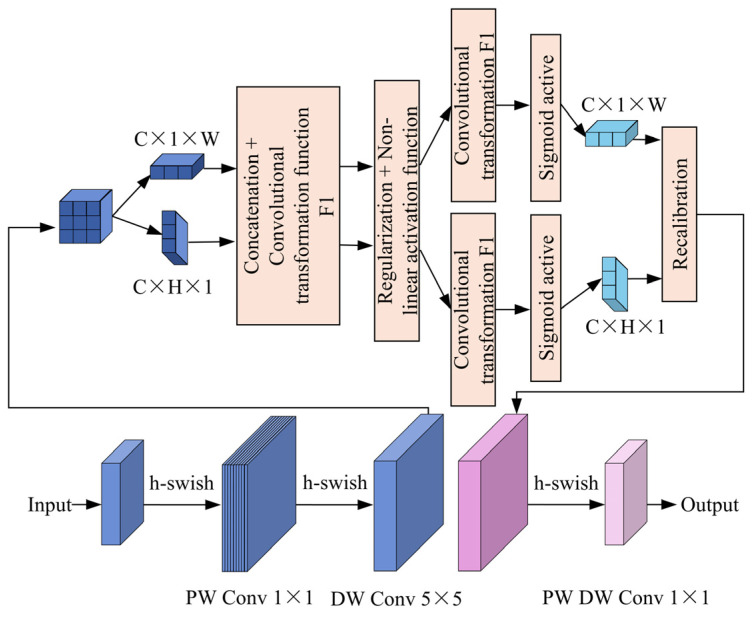
Bottleneck structure of the MobileNetV3-CA network. The upper part illustrates the coordinate attention (CA) mechanism, which encodes spatial information along both height and width directions and performs feature recalibration. The lower part shows the standard bottleneck structure with pointwise and depthwise convolutions, as well as h-swish activation functions.

To meet the stringent requirements for real-time detection of small defects on rail surfaces in industrial settings, this paper proposes a lightweight reconstruction of the DeepLabv3+ backbone network. Although the traditional Xception network [27] exhibits strong feature representation capabilities in complex, textured backgrounds, its complex architecture, high parameter count, and substantial computational demands impede efficient deployment and real-time performance, particularly on resource-constrained industrial devices. To address these challenges, this paper integrates MobileNetV3 with the coordinate attention mechanism, optimizing computational efficiency and detection accuracy. The architecture of the modified bottleneck structure is depicted in Figure 2.

In the feature extraction stage, this paper presents an optimization of the inverted residual structure in MobileNetV3. Specifically, the standard 3 × 3 convolution is replaced with a 5 × 5 depthwise separable convolution, utilizing an expansion factor of 2. This modification preserves the receptive field while enhancing the efficiency of information fusion across channels, achieving a balanced compromise between computational efficiency and feature representation capacity. To further improve the model’s deployment efficiency on embedded platforms, the conventional ReLU activation function has been replaced with the h-swish activation function. The definition of the h-swish activation function is provided in Equation (1):

(1) $$h-swish\left[x\right]=x\frac{ReLU6\left(x+3\right)}{6}$$

ReLU6 is defined as follows:

(2) $$ReLU6=min(6,max\left(0,x\right))$$

The h-swish function maintains the smooth characteristics of the Swish activation while incorporating ReLU6 to diminish computational demands. This adaptation also sustains gradient continuity near zero, facilitating more effective feature correlation capture. Consequently, this configuration substantially reduces the computational burden, rendering it ideal for deployment on industrial devices with limited resources.

However, the conventional SE attention mechanism [28] excels at enhancing feature representation in the channel dimension but has limited capacity for capturing spatial information. Surface defects on rails typically exhibit diverse forms and complex spatial distributions. For example, slender scratches may extend longitudinally across the rail surface, while local pits may be concentrated in specific regions. To more effectively capture the spatial characteristics of these defects, this paper introduces the coordinate attention mechanism as a replacement for the original SE attention mechanism. The coordinate attention mechanism integrates spatial coordinate information into the attention weights, thereby enhancing the model’s ability to capture spatial location details while preserving global information. The coordinate attention mechanism involves two critical stages: coordinate information embedding and coordinate attention generation.

In the coordinate information embedding phase, the model compresses the spatial dimensions (H × W) of the feature map into two independent one-dimensional vectors through global pooling operations. These vectors represent the global information along the height and width directions, respectively. Given that the input feature map is denoted as X∈R^C×H×W^, where C is the number of channels, and H and W represent the height and width of the feature map, respectively, the pooling operation along the height direction is expressed as Equation (3):

(3) $$z_{h}\left(h\right)=\frac{1}{W}\sum_{\omega =1}^{W}X(h,\omega )$$

The pooling operation along the width direction is represented by Equation (4):

(4) $$z_{\omega }\left(\omega \right)=\frac{1}{H}\sum_{h=1}^{H}X(h,\omega )$$

It is noted here that the one-dimensional vectors z_h_ and z_ω_ capture the global information of the feature map along the height and width directions, respectively.

In the subsequent stage of coordinate attention generation, the vectors z_h_ and z_ω_ are used to generate attention weights through convolution and non-linear transformation. These weights are then applied to the height and width dimensions of the feature map. Specifically, the following steps are performed: First, A and B are concatenated. Then, attention weights are generated via a 1 × 1 convolutional layer followed by a non-linear activation function (e.g., Sigmoid), as described in Equation (5):

(5) $$A_{h}=\sigma (F_{h}\left(z_{h}\right)),A_{\omega }=\sigma (F_{\omega }\left(z_{\omega }\right))$$

Here, F_h_ and F_ω_ represent the convolution operations along the height and width directions, respectively, while σ denotes the Sigmoid function. Finally, the attention weights A_h_ and A_ω_ are multiplied by the corresponding input feature map, yielding the weighted feature map, as expressed in Equation (6):

(6) $$Y(h,\omega )=X(h,\omega )\cdot A_{h}\left(h\right)\cdot A_{\omega }\left(\omega \right)$$

### 3.2. Dual-Stream Feature Fusion Module

In the task of detecting surface defects on rails, traditional deep learning methods often struggle with accurately segmenting defect regions. This challenge arises primarily from the complexity of the image background and the low contrast of defect regions. To overcome these technical difficulties, this paper introduces a surface defect detection approach that leverages the fusion of RGB and polarization image bimodalities. This fusion is predicated on the assumption that it significantly enhances the contrast between material surfaces and defects, thus substantially improving the accuracy and robustness of surface defect segmentation.

The enhanced DeepLabV3+ model introduces a dual-branch feature extraction network, employing two parallel MobileNetV3-CA backbones—one for RGB and the other for polarization images. This architecture not only captures distinct color and texture features from RGB images but also extracts polarization characteristics and material properties, thereby enriching the visual information. The integration of these diverse features into subsequent network layers ensures a comprehensive and effective representation.

**Figure 3:**
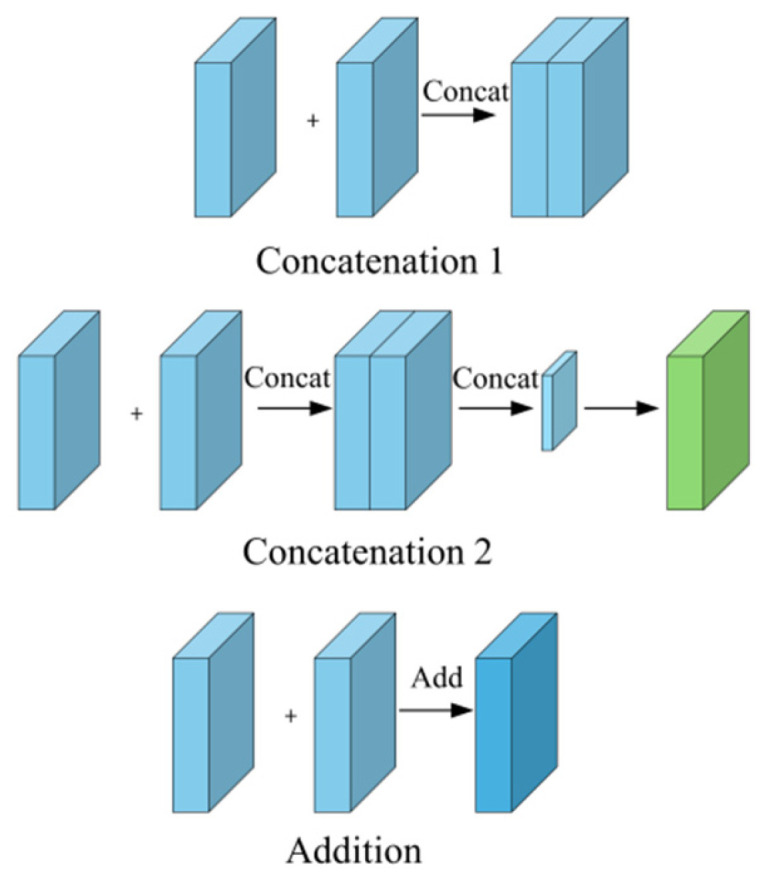
Illustration of three feature fusion methods: (1) concatenation 1, (2) concatenation 2, and (3) pixelwise addition. For clarity, the “addition” operation refers to pixelwise addition of feature maps.

Following the extraction of features, this paper introduces three methods for fusing the obtained light intensity and polarization features, as depicted in Figure 3. After rigorous testing, it was found that fusing the feature maps from the two branches via pixelwise addition yielded the most optimal results. This fusion approach effectively preserves the complementary information from both the RGB and polarization images. Specifically, it retains color and texture information from the RGB images, as well as material and surface property details from polarization images. Moreover, this method minimizes feature redundancy, ensuring a more holistic representation for subsequent defect segmentation tasks. Additionally, the pixelwise addition operation has relatively low computational complexity, making it ideal for real-time deployment and meeting the demands of practical applications.

After feature fusion, the resulting feature maps are passed into the Atrous Spatial Pyramid Pooling (ASPP) module of DeepLabV3+. This module effectively captures multi-scale contextual information, thereby expanding the model’s receptive field. Through operations such as cascaded upsampling and skip connections, the spatial resolution of the feature maps is progressively restored, ultimately producing high-precision defect segmentation results. This design enables the model to fully exploit the advantages of multimodal features, achieving accurate defect detection even in complex backgrounds.

### 3.3. CBAM Decoder

In the original DeepLabV3+ model, the decoder uses two 4× upsampling operations to restore the feature map to the size of the input image. However, this high-magnification upsampling may result in a significant loss of pixel information. Although the feature map from the encoder is concatenated and fused at the decoder’s quarter position to bolster feature information, this process can introduce noise and potentially reduce segmentation accuracy. To address this challenge, we adopt the widely used progressive upsampling strategy [29,30,31], replacing the conventional 4× upsampling with two consecutive 2× upsampling operations. This approach, commonly used in semantic segmentation and super-resolution tasks, helps reduce the computational load on the upsampling modules. This change not only allows for a more gradual and controlled expansion of feature maps, reducing the risk of losing fine details during upscaling, but also facilitates better integration of multi-scale features, enhancing the overall accuracy of defect segmentation.

**Figure 4:**
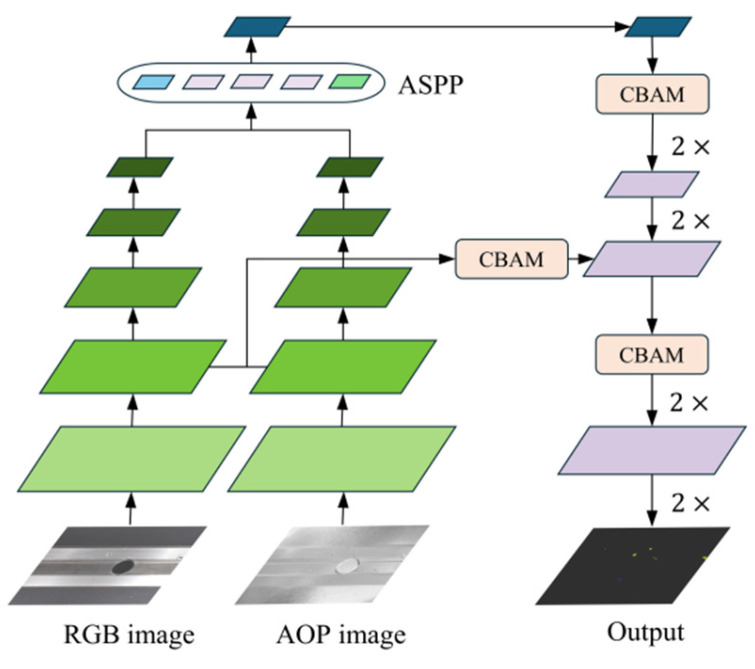
Architecture of CBAM decoding. The network takes both RGB and AOP images as input, extracts and fuses multi-scale features through an encoder and ASPP module, and then progressively decodes them with the aid of the CBAM to generate the final output.

Additionally, to further enrich feature information and effectively reduce the impact of noise on segmentation accuracy, this paper introduces the Convolutional Block Attention Module (CBAM). The CBAM combines channel and spatial attention mechanisms to enhance the feature fusion process, both before and after the integration of deep and shallow feature layers. Comprising channel attention and spatial attention, the CBAM allows the network to focus on critical regions during feature extraction. This prioritization significantly improves the network’s capacity to capture detailed information on small-target defects and boosts the overall accuracy of the segmentation process. The enhanced CBAM decoding structure is illustrated in Figure 4.

Furthermore, the channel attention module utilizes global average pooling and global maximum pooling to extract statistical information from each channel. These data are processed through two fully connected layers to determine the channel weights. The combined outputs are then passed through a Sigmoid activation function to calculate the final channel attention weights. Finally, the attention weights are applied to the feature map F, resulting in the output feature map F’, as expressed in Equation (7):

(7) $$M_{c}\left(F\right)=\sigma \left(MLP\left(AvgPool\left(F\right)\right)+MLP\left(MaxPool\left(F\right)\right)\right)=\sigma \left(W_{1}\left(W_{0}\left(F_{avg}^{c}\right)\right)+W_{1}\left(W_{0}\left(F_{max}^{c}\right)\right)\right)$$

where MLP refers to a multi-layer perceptron, σ represents the activation function, W_0_ and W_1_ denote the weights, AvgPool stands for average pooling, and MaxPool denotes maximum pooling. Additionally, F^C^_avg_ and F^C^_max_ represent the elements of average pooling and maximum pooling, respectively. It is noted here that integrating this module into the model improves its ability to focus on critical channels, thereby enhancing the feature map’s capacity to highlight and represent essential regions of information.

Moreover, the spatial attention module employs both maximum and average pooling operations to derive the maximum and average values at each spatial position. These operations yield two distinct feature matrices that are subsequently concatenated. The weight distribution of the learning space position is determined through a convolution layer followed by a Sigmoid function. Finally, the model multiplies the spatial attention weights with the input feature map to generate the weighted output feature map, as described in Equation (8):

(8) $$M_{s}\left(F\right)=\sigma \left(f^{7*7}\left(\left[AvgPool\left(F\right);MaxPool\left(F\right)\right]\right)\right)=\sigma \left(f^{7*7}\left(\left[F_{avg}^{s};F_{max}^{s}\right]\right)\right)$$

where F^s^_avg_ and F^s^_max_ represent the average and maximum pooling features, respectively, across channels. The f^7∗7^ indicates the convolution operation performed with a 7∗7 convolution kernel. This module enhances the model’s ability to identify and prioritize important regions in the spatial dimension, thereby improving segmentation performance in complex scenarios.

## 4. Experiment and Result Analysis

### 4.1. Experimental Environment

The experiments were conducted using the PyTorch1.13.1 framework, with the MM-Segmentation platform serving as an extension library. All training was performed on a system equipped with an RTX 4090 Ti GPU (24 GB VRAM) (NVIDIA Corporation, Santa Clara, CA, USA) in a Python 3.9 environment using the PyCharm3.9 IDE. The batch size was set to 8, and model training was carried out for 50,000 iterations. The AdamW optimizer was selected for its effectiveness in mitigating overfitting. A Poly learning rate schedule was adopted, with the initial learning rate set to 0.0001 and dynamically adjusted throughout the training process. Additionally, a weight decay coefficient of 0.05 was applied to further regulate the model’s complexity.

**Figure 5:**
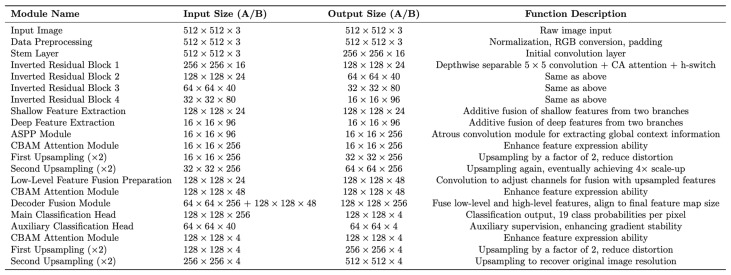
Shape and functional description of the feature maps processed at each step.

In addition, we provided detailed computational procedures for each stage of our proposed model for supporting high reproductivity. Specifically, the model received two input images of size 512 × 512 × 3, which were preprocessed and passed through an initial convolutional layer (stem layer) before entering the backbone network, composed of inverted residual blocks. These blocks utilize 5 × 5 depthwise separable convolutions with an expansion factor of 2, combined with a coordinate attention (CA) mechanism and the h-swish activation function, to extract multi-scale features. During feature extraction, shallow features (128 × 128 × 24) and deep features (16 × 16 × 96) are extracted from two branches and fused using an additive strategy. The fused deep features are then fed into an ASPP module, which employs multi-scale dilated convolutions to extract global contextual information. This is followed by a CBAM to enhance feature representation. In the decoder, two consecutive 2× upsampling operations gradually restore the spatial resolution of feature maps. Three CBAMs are embedded throughout the decoder, both before and after feature fusion, to further improve feature expression. Finally, the main classification head generates a per-pixel probability map for four classes, which is upsampled twice to match the original image’s resolution. An auxiliary classification head provides additional supervision during training, facilitating gradient propagation and improving the training process. The shape and functional description of the feature maps processed at each step are shown in Figure 5 below.

### 4.2. Dataset

#### 4.2.1. RGB Dataset

**Figure 6:**
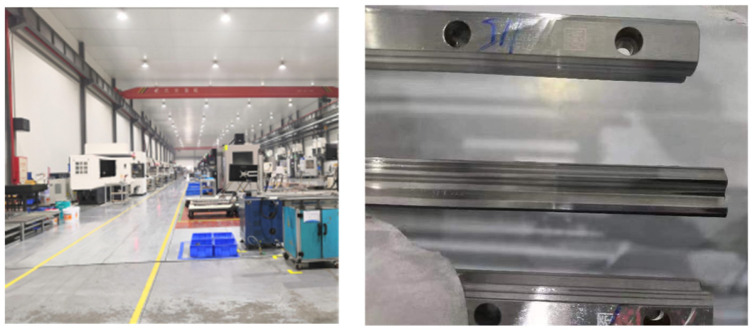
Data collection and experimental space.

In this study, the data were collected from the primary production line at the Guangdong Kite Precision Machinery Co., Ltd. (Dongguan, China), between October and December of 2022. It includes data from multiple batches of rail production lines, as shown in Figure 6. A total of 800 original sample sets were acquired using a high-precision industrial camera system. After thorough screening by a professional quality inspection team, based on criteria such as image clarity, defect integrity, and annotation accuracy, 720 effective samples were selected to create the standard dataset. These samples are evenly distributed across various workstations and stages of the production process, ensuring that the data are both representative and diverse.

**Figure 7:**
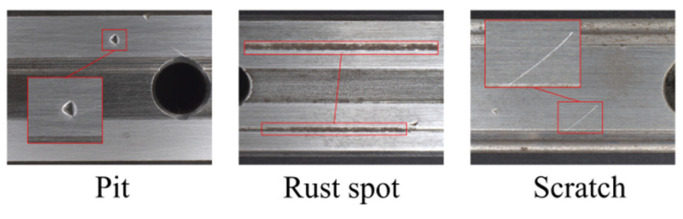
Example of rail surface defects.

It is worth noting here that the collected images have a high resolution of 2048 × 1536 pixels, providing a clear view of the microscopic features of the rail surface. Additionally, the spatial proportion of the defect regions in the images varies from 0.05% to 5.12%, making the dataset suitable for identifying small defects in industrial inspection applications. The dataset focuses on three common types of defects on the rail surface: scratches (linear surface damage), rust spots (oxidation corrosion areas), and pits (mechanical impact marks). The dataset statistics are shown in Table 1, some examples as shown in Figure 7.

In addition, Figure 5 offers a detailed analysis of the morphological characteristics of each defect type, serving as a reliable benchmark for future algorithmic research.

#### 4.2.2. Polarization Defect Dataset

**Figure 8:**
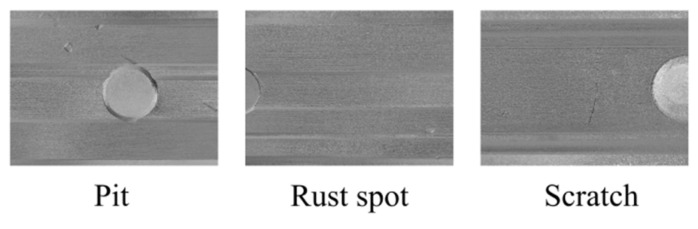
Polarization defect image.

Polarization images were acquired synchronously using polarization imaging technology to capture a polarization optical defect dataset aimed at multi-physical feature characterization. This process was based on the existing RGB defect dataset for rails, as illustrated in Figure 8. Unlike traditional RGB images, which capture only the light intensity distribution, polarization imaging analyzes the Stokes vector parameters of reflected light—a mathematical representation of the polarization state of light that includes information about total intensity, linear polarization components, and circular polarization. This analysis enables the extraction of additional features, such as the angle of polarization (AoP) and the degree of linear polarization (DoLP). Polarization features are highly sensitive to surface micro-deformations, particularly at the transition between specular and diffuse reflection, significantly enhancing the optical response to low-contrast defects. By integrating polarization features with the RGB appearance data, this approach enables a multidimensional characterization of industrial defects, including scratches, rust, dents, and stains. This fusion provides a more comprehensive and informative foundation for the subsequent analysis of both optical and structural characteristics.

To ensure effective cross-modal feature learning, polarization and RGB data were meticulously aligned in both spatial and temporal dimensions. The process is as follows: **(1) Spatial Alignment**: We performed geometric calibration using a standard checkerboard to estimate the transformation between RGB and polarization images. All images were then spatially registered using this transformation to ensure pixel-level correspondence. **(2) Temporal Alignment**: We synchronized image acquisition through hardware triggers when possible. If not, we matched image pairs based on timestamps, selecting the closest frames from both modalities to minimize temporal offset. For data partitioning, stratified random sampling was employed for both datasets to create training and test sets in a 4:1 ratio. The training set was further enhanced through data augmentation techniques, including adaptive lighting transformations, non-rigid deformation, multi-scale Gaussian blurring, and composite noise injection. These augmentations are designed to simulate complex industrial lighting conditions and imaging interferences. The statistical information of the augmented dataset used in our experiments is presented in Table 2. The dataset construction process carefully addresses the inherent challenges of industrial environments, such as high-reflectivity surfaces, textured background interference, and multi-scale defects. As a result, this dataset provides a robust validation platform for deep learning models, offering a realistic simulation of industrial scenarios.

### 4.3. Evaluation Metrics

In the experimental validation phase, this study utilized four core metrics—mIoU (mean intersection over union), mPrecision (mean precision), mRecall (mean recall), and F1-score—to assess model performance. The mIoU metric quantifies spatial segmentation accuracy by measuring the overlap between predicted regions and the corresponding ground truth annotations. This metric evaluates the model’s ability to differentiate between foreground and background objects. The mPrecision metric gauges the reliability of the model’s positive predictions, reflecting its effectiveness in minimizing false positives. On the other hand, the mRecall metric measures the model’s ability to identify all relevant positive samples, demonstrating its capacity to avoid missed detections. The F1-score serves as a harmonic mean of the precision and recall metrics, offering a comprehensive evaluation of the model’s overall classification performance by balancing both metrics. The mathematical formulas for each of these metrics are outlined in Equations (9)–(12).

(9) $$mIoU=\frac{1}{n_{cls}}\cdot \sum_{i}\frac{n_{ii}}{\sum_{j}n_{ij}+\sum_{j}n_{ji}-n_{ii}},$$

(10) $$\begin{array}{c} \mathrm{mPrecision}=\frac{1}{n_{cls}}\cdot \sum_{i}\frac{n_{ii}}{\sum_{j}n_{ji}}\; \end{array},$$

(11) $$\begin{array}{c} \mathrm{mRecall}=\frac{1}{n_{cls}}\cdot \sum_{i}\frac{n_{ii}}{\sum_{j}n_{ij}} \end{array}$$

(12) $$\begin{array}{c} F1=\frac{2\times Precision\times Recall}{Precision+Recall} \end{array}$$

where $n_{cls}$ represents the number of classes, and $n_{ij}$ denotes the number of pixels from class i predicted as class j.

### 4.4. Performance Comparison

To assess the performance of the enhanced DeepLabv3+ model in surface defect segmentation of rails, the proposed DIR-DeepLabv3+ model was compared with several benchmark models, including UNet [32], KNet [33], PSPNet [34], and the original DeepLabv3+ model. The results of this comparison, showcasing the performance of each model in the rail surface defect segmentation task, are presented in Table 3.

As shown in Table 3, our proposed model outperforms other comparative models in complex detection environments while also achieving the highest running efficiency (FPS). Specifically, the proposed model outperforms the KNet model, which demonstrates the best performance among the most comparative models. Despite utilizing only 40.44% of KNet’s parameters, the proposed model achieves a 1.5% higher mean intersection over union (mIoU) and a 0.49% higher pixel accuracy in rail segmentation tasks. When compared to UNet, which has a similar parameter count, the proposed model shows a 3.32% increase in mIoU and a 1.39% increase in pixel accuracy. In contrast to the PSPNet model, which has a significantly lower parameter count, the proposed model achieves improvements of 6.04% in mIoU and 1.07% in pixel accuracy. While PSPNet’s modest number of parameters and simpler architecture are advantageous, its performance in segmenting complex rail surface defects remains suboptimal, with challenges in balancing accuracy and processing speed.

Furthermore, when compared to DeepLabV3+, the proposed model demonstrates a 3.23% increase in mIoU and a 1.21% improvement in pixel accuracy, while simultaneously reducing floating-point operations by 42.94%. This suggests that by incorporating the lightweight MobileNetV3 backbone, the model’s inference speed is notably accelerated. Overall, the proposed model strikes an optimal balance between parameter count, performance, computational efficiency, and segmentation accuracy. It not only outperforms other models in terms of real-time processing but also delivers superior segmentation accuracy, reaffirming its effectiveness in complex industrial defect detection tasks.

**Figure 9:**
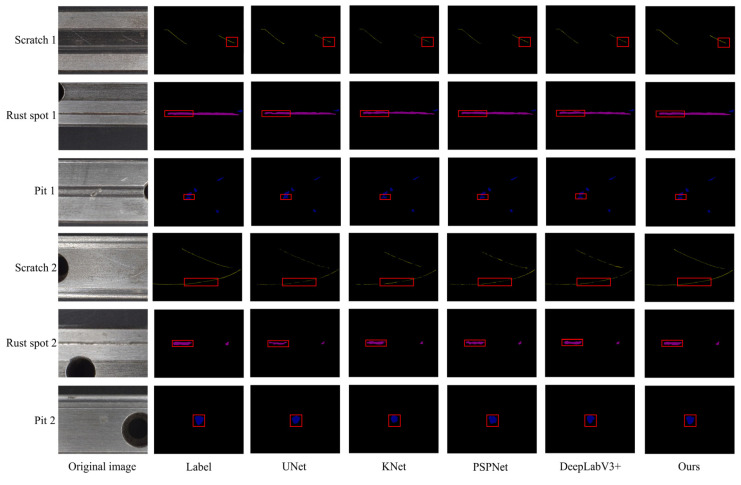
Visualization of segmentation results for rail surface defects using different models. The red boxes highlight the regions of interest where defects are present.

As illustrated in Figure 9, two sets of images from the test set, each representing one of the three defect types, were randomly selected for a visual comparison of the segmentation results from various models. This method clearly demonstrates the effectiveness of the proposed model in detecting surface defects on rails.

The first three rows display the initial set of images for each defect category, while the following three rows present the second set. In the first and second columns, the original images of the rail surface defects and their corresponding labels are shown. The third to seventh columns present the prediction results from the comparison models, while the eighth column showcases the results from the proposed model.

As illustrated in Figure 9, the prediction results for slender scratches in the first and fourth rows reveal that the other four comparison models miss significant portions of the scratches, while the proposed DIPF_DeepLabv3+ accurately predicts the defects. Even the UNet model, which generally performs well in segmentation tasks, still exhibits some false positives and missed detections, with extra segmented areas appearing at the start and end of the scratches. The PSPNet model, despite its efficiency, struggles with segmentation area deviations and has difficulty accurately identifying scratches amidst complex textures. The third row, showing the pits, highlights the challenge of accurately segmenting defects with high density and similar texture and color to the background. It is noted that all other comparison models fail to achieve accurate segmentation. In contrast, DIPF_DeepLabv3+ stands out as the only model capable of effectively distinguishing inter-class differences and accurately segmenting the densely packed pits. This result indicates that the proposed DIPF_DeepLabv3+ model excels in learning and extracting defect features, making it highly effective at segmenting low-contrast surface defects on rails. Furthermore, the results from the remaining rows highlight that DIPF_DeepLabv3+ demonstrates superior precision in identifying and segmenting rail surface defects.

### 4.5. Ablation Experiment

In the proposed DIPF_DeepLabv3+ model, the MobileNetV3-CA backbone network module (M1) addresses the challenges of complex network structures and high computational demands. The dual-stream feature information fusion module (M2) is designed to mitigate recognition difficulties caused by low defect contrast in images. Meanwhile, the CBAM decoder (M3) aims to improve segmentation accuracy for small-sized defects. To evaluate the contribution of each module, this study conducted an ablation experiment with seven ablation settings, analyzing the effectiveness of the proposed M1, M2, and M3 modules. Among the seven ablation experiments, the first corresponds to the baseline DeepLabV3+ model, and the seventh corresponds to the DIPF_DeepLabv3+ model. The complete results of the ablation study on the rail defect dataset are presented in Table 4, which systematically documents the incremental enhancements in the model’s segmentation performance. The values in parentheses represent the enhancement relative to the baseline model. The symbol “×” indicates that a given module is not included, while “√” indicates that the module is incorporated. This notation method provides a clear and intuitive demonstration of how different module combinations influence the overall model performance.

In Experiment 2, replacing the original Xception network in the DeepLabv3+ model with MobileNetV3-CA as the backbone network results in a significant reduction in model parameters. This not only decreases computational complexity but also greatly improves testing speed. It is noted that while the model is becoming more lightweight, segmentation accuracy improves slightly compared to the original Xception network, highlighting the advantages of Mobilenetv3-CA in feature extraction. Experiments 3 and 4 demonstrate that incorporating the dual-stream feature fusion module and the CBAM decoder into the DeepLabv3+ model, respectively, further enhances its performance. Experimental results in Table 3 show improvements in two key evaluation metrics, mIoU and mF_1_, indicating the effectiveness of these introduced modules. Replacing the Xception backbone with MobileNetV3-CA reduces model complexity while slightly improving segmentation accuracy, showcasing its superior feature extraction capabilities. These further demonstrate the effectiveness and superiority of our MobileNetV1-CA architecture in potential representation learning.

In Experiment 5, building upon the foundation of Experiment 2, a dual-stream feature information fusion module is introduced. This module benefits from a dual-backbone network that extracts features at multiple scales, capturing both global context and local details more efficiently. Consequently, the mIoU and F1 scores increase by 2.93% and 1.15%, respectively. Additionally, in Experiment 6, the CBAM decoder is added to the model developed in Experiment 2. This slight increase in computational load led to the enhanced ability of the model to emphasize effective channel features, thereby strengthening its capacity to encode rich high-level information. This results in a 1.84% increase in mIoU. Finally, in Experiment 7, the dual-stream feature information fusion module from Experiment 6 is further enhanced by introducing two branches dedicated to extracting feature information from RGB and polarization images. Thus, refinement significantly improves the extraction of complex details related to rail defects, resulting in a notable 3.23% increase in the mIoU. This indicates that the dual-stream feature fusion module provides the CBAM decoder with richer feature information, enabling the CBAM decoder to focus more effectively on critical features. The two components complement each other, jointly enhancing the model’s performance. These results further demonstrate the effectiveness of our proposed method. In addition, in the ablation studies of the three core modules (M1, M2, M3), we observe a decrease in FPS when any module is removed. This further demonstrates the effectiveness and necessity of each module in enhancing the real-time performance of our model.

The proposed model in this study replaces the original backbone network with MobileNetV3-CA, resulting in significant reductions in both model parameters and floating-point operations, thus achieving a lighter model. Simultaneously, the integration of two additional modules substantially enhances the model’s segmentation accuracy and overall performance. Compared to the baseline model, the model’s mIoU and mean F1 score increase by 3.23% and 1.61%, respectively, while using only 68.45% of the original parameters. This improvement highlights the effectiveness of the revised model architecture, leading to a marked reduction in both storage and computational resource requirements. Moreover, the model achieves lightweighting by striking an effective balance between segmentation accuracy, model complexity, and computational efficiency. The three modules complement and reinforce each other, contributing synergistically to the final model’s performance. As a result, the proposed DIPF_DeepLabv3+ model demonstrates the ability to accurately detect and segment small, low-contrast defects, attaining advanced performance on the rail surface defect dataset.

## 5. Discussion

For the issue of dataset limitation, we acknowledge that our dataset consists of 720 samples, with a relatively small proportion of defect cases. While the dataset size is limited, we would like to emphasize that all samples were collected from real-world industrial scenarios (as shown in Figure 6), which ensured the authenticity and practical relevance of our data. Despite the limited data size, the models trained on this dataset have already been successfully deployed in real industrial environments and have demonstrated strong performance in practical applications. This indicates the robustness and practical value of our approach, even under real-world constraints. In addition, despite the overall strong performance of our proposed model, certain defect types are more challenging for the model and can occasionally lead to missed or false detections. Specifically, defects with extremely subtle features or those that are visually like normal regions may be more difficult to identify accurately. For the real-time deployment constraint, we added new metrics of FPS in the revised manuscript that indicate that our model can achieve the highest 29.63 FPS with 24 GB VRAM in real-world deployments (as shown in Table 3), demonstrating its real-time effectiveness. Importantly, we would like to highlight that, even with these challenges, our approach has demonstrated robust and reliable results in real-world industrial deployment. This practical success underscores the effectiveness and applicability of our method.

## 6. Conclusions and Future Work

This paper introduces a method for lightweight, precise segmentation and identification of low-contrast, small-target defects on rail surfaces against complex backgrounds. The proposed method employs MobileNetV3 as a lightweight backbone, replacing the original feature extraction network and incorporating the channel attention (CA) mechanism to markedly reduce model complexity and enhance spatial perception. The encoder introduces a dual-stream feature information fusion module to adaptively combine features from both RGB and polarization images, thereby improving the segmentation of low-contrast defects. In the decoder, the implementation of the CBAM effectively processes both shallow and deep features, enhancing the model’s ability to detect defect edge details and increasing the accuracy of small-target defect segmentation. Experimental results demonstrate that the enhanced model achieves significant improvements in both lightweight design and segmentation accuracy. The model’s parameter count is reduced to 28.21 M, a 31.55% decrease from the original model, while floating-point operations are cut by 42.94%. The mean intersection over union (mIoU) metric impressively reaches 86.21%, and pixel segmentation accuracy rises to 98.91%. The proposed model skillfully balances a lightweight design with improved segmentation accuracy. Future research should concentrate on refining the model’s lightweight architecture and investigating fusion strategies for multi-scale, high-resolution features to further enhance segmentation accuracy and generalization across various rail surface defect segmentation tasks.

## Figures and Tables

**Table 1:** Dataset statistics of the used dataset.

Defect Type	Number of Data
Pits	215
Rust spots	145
Scratches	360
Total	720

**Table 2:** Detailed statistic information about the dataset we used in this paper.

Dataset	Original Data	Augmented Data	Training Data	Test Data
**RGB**	720	2160	1728	432
**Polarization**	720	2160	1728	432

**Table 3:** Performance comparison of defect detection across different models. Bold: the best; underline: the second best.

Model	IoU of Each Defect Label				mIoU	m-Pre	m-Re	Flops (GB)	Param (M)	F1	FPS
	Background	Scratch	Rust Spot	Pit							
UNet	99.56	67.45	76.86	90.33	83.55	90.14	91.94	182.93	**24.89**	91.03	18.76
KNet	99.66	69.26	77.63	94.93	85.37	91.04	91.69	281.28	69.76	91.36	24.61
PSPNet	99.56	63.76	72.54	87.46	80.83	90.46	87.74	18.8	105.38	89.08	13.12
DeepLabV3+	99.63	68.53	77.21	89.19	83.64	90.32	90.58	67.54	41.21	90.47	16.64
**Ours**	**99.86**	**72.64**	**78.31**	**96.97**	**86.87**	**91.53**	**93.14**	38.54	28.21	92.33	**29.63**

**Table 4:** Comparative results of ablation experiments for the DIPF_DeepLabv3+ model. ✓ denotes the evaluation is carry the specific module (i.e., M1, M2, and M3).

Experiment No.	M1	M2	M3	mIoU (%)	mF_1_ (%)	Flops (GB)	Params (M)	FPS
1				83.64	90.72	67.54	41.21	16.64
2	✓			83.79 (+0.15)	91.29 (+0.57)	19.61	17.82	21.34
3		✓		86.39 (+2.75)	91.36 (+0.64)	167.23	74.71	10.38
4			✓	85.07 (+1.43)	91.57 (+0.85)	73.22	46.29	13.25
5	✓	✓		86.57 (+2.93)	91.87 (+1.15)	35.83	23.22	23.23
6	✓		✓	85.48 (+1.84)	92.05 (+1.33)	21.22	22.80	24.72
7	✓	✓	✓	86.87 (+3.23)	92.33 (+1.61)	38.54	28.21	29.63

## Data Availability

The raw data supporting the conclusions of this article will be made available by the authors on request.
